# Altered Metabolism of Phospholipases, Diacylglycerols, Endocannabinoids, and *N*-Acylethanolamines in Patients with Mastocytosis

**DOI:** 10.1155/2019/5836476

**Published:** 2019-07-01

**Authors:** Anne Lise Ferrara, Fabiana Piscitelli, Angelica Petraroli, Roberta Parente, Maria Rosaria Galdiero, Gilda Varricchi, Giancarlo Marone, Massimo Triggiani, Vincenzo Di Marzo, Stefania Loffredo

**Affiliations:** ^1^Department of Translational Medical Sciences and Center for Basic and Clinical Immunology Research (CISI), University of Naples Federico II, WAO Center of Excellence, Naples, Italy; ^2^Endocannabinoid Research Group, Istituto di Chimica Biomolecolare-Consiglio Nazionale delle Ricerche (ICB-CNR), Pozzuoli, Italy; ^3^Division of Allergy and Clinical Immunology, University of Salerno, Italy; ^4^Department of Public Health, University of Naples Federico II, Italy; ^5^Monaldi Hospital Pharmacy, Naples, Italy; ^6^Canada Excellence Research Chair on the Microbiome-Endocannabinoidome Axis in Metabolic Health, Université Laval, Centre de Recherche de l'Institut Universitaire de Cardiologie et Pneumologie de Quèbec, and Institut sur la Nutrition et les Aliments Fonctionnels, Québec City, Canada

## Abstract

**Background:**

Mastocytosis is a condition characterized by the expansion and accumulation of mast cells (MCs) in various organs. The symptoms are related to the increased release of MC-derived mediators that exert local and distant effects. MCs are a source and target of phospholipase enzymes (PLs), which catalyze the cleavage of membrane phospholipids releasing lipid mediators (e.g., diacylglycerols (DAGs) and the endocannabinoid (EC) 2-arachidonoylglycerol (2-AG)). To date, there are no data on the role of these lipid mediators in mastocytosis. Here, we analyzed plasma levels of PLA_2_, PLC, DAG, ECs, and EC-related *N*-acylethanolamines in patients with mastocytosis.

**Methods:**

In 23 patients with mastocytosis and 23 healthy individuals, we measured plasma PLA_2_ and PLC activities, DAG, 2-AG, anandamide (AEA), palmitoylethanolamide (PEA), and oleoylethanolamide (OEA).

**Results:**

Plasma PLA_2_ and PLC activities were increased in mastocytosis patients compared to controls. Concentrations of DAG (18:1 20:4 and 18:0 20:4), two second messengers produced by PLC, were higher in mastocytosis compared to controls, whereas the concentrations of their metabolite, 2-AG, were not altered. AEA was decreased in mastocytosis patients compared to controls; by contrast, AEA congener, PEA, was increased. PLA_2_ and PLC activities were increased only in patients with mediator-related symptoms. Moreover, PLC activity was positively correlated with disease severity and tryptase concentrations. By contrast, AEA was negatively correlated with tryptase concentrations.

**Conclusions:**

PLs and some lipid mediators are altered in patients with mastocytosis. Our results may pave the way for investigating the functions of these mediators in the pathophysiology of mastocytosis and provide new potential biomarkers and therapeutic targets.

## 1. Introduction

Mastocytosis is a disease characterized by the abnormal proliferation and/or accumulation of clonal mast cells (MCs) in the skin and other organs [1]. The pathogenesis of mastocytosis is related to an activating mutation of the KIT receptor localized on MCs, which leads to uncontrolled MC proliferation [2].

Patients with mastocytosis can be classified into two main groups characterized by different clinical courses and prognosis: cutaneous mastocytosis (CM) and systemic mastocytosis (SM) [3]. In CM, MC accumulation is limited to the skin, whereas in SM, at least one extracutaneous tissue is involved. The variants of mastocytosis are shown in Supplementary Table 1 [4]. In the majority of patients with mastocytosis, symptoms are due to the activation and degranulation of MCs and are the consequences of their local or systemic effects [5]. Mediator-related symptoms and clinical signs are found in all variants of mastocytosis and may involve different organ systems [6].

MCs produce a plethora of preformed (histamine, tryptase, etc.) and *de novo* synthesized (lipids, cytokines, etc.) mediators, which exert different biological effects [7, 8]. Activated MCs express and release phospholipase enzymes (PLs) which catalyze the cleavage of membrane phospholipids [9, 10]. There are four classes of phospholipases termed A (PLA), B (PLB), C (PLC), and D (PLD) [11–13], distinguished by substrate specificity, subcellular location, and functional importance of their phospholipid metabolites. Enzymatic processing of phospholipids by PLs converts them into lipid mediators or second messengers (such as diacylglycerols (DAGs), endocannabinoids (ECs), and arachidonic acid (AA)), which activate effector enzymes (such as protein kinase C (PKC)) and regulate multiple cellular processes of several cells including MCs [14–16].

Secreted PLA_2_ (sPLA_2_s), expressed by MCs, are released into the extracellular fluid upon cellular activation and modulate cell degranulation [9, 17–19]. This feature of sPLA_2_s explains their presence in biological fluids of patients with inflammatory diseases including asthma, autoimmune diseases, allergic diseases, and cancer [20–25]. sPLA_2_s can exert their function through cleavage of membrane phospholipids or via receptors [26–29]. Murakami et al. reported that the blocking of the heparin-binding domain of sPLA_2_ suppresses PLA_2_ group IIA-induced histamine release in murine mast cells [30]. The sPLA_2_s are essential for the release of AA from phospholipids and, thereby, for the production of eicosanoids that are produced in large quantities in patients with mastocytosis [31–34].

PLC together with PLD are essential signals for MC activation and degranulation [10, 35–37]. Hydrolysis of phosphatidylinositol 4,5-bisphospate by PLC, and of phosphatidylcholine by PLD followed by the action of phosphatidic acid hydrolase, is the major source of DAGs in stimulated MCs [14, 38, 39]. DAGs are physiological activators of PKC, and in the case of *sn*-2-arachidonoyl-DAG species, they are also precursors of the endocannabinoid 2-arachidonoylglycerol (2-AG) through the action of DAG lipases (DAGLs). Apart from acting on cannabinoid receptors, 2-AG can also be an alternative precursor of AA and eicosanoids [14, 40].

The ECs 2-AG and anandamide (AEA), together with non-EC AEA congeners, i.e., *N*-acylethanolamines like oleoylethanolamide (OEA) and palmitoylethanolamide (PEA), are biosynthesized “on demand” from membrane phospholipids and modulate the functional activities of a variety of cells including MCs [41–43]. However, unlike 2-AG, *N*-acylethanolamines are produced from the action serine hydrolases different from PLs [44]. Yet, PEA possess the ability to reduce both acute and chronic inflammations by downmodulating activated MCs [16, 43, 45–48]. MCs express cannabinoid receptors (CB) [49] that regulate MC activation [43]. Indeed, CB2 activation by 2-AG and AEA downregulates MC degranulation [50, 51].

Owing to the ability of PLs, DAGs, ECs, and *N*-acylethanolamines to modulate MC biology (either by directly activating MCs or by catalyzing the production/degradation of other molecules), we have analyzed the plasma concentration or activity of these lipid mediators in patients with mastocytosis.

## 2. Methods

### 2.1. Study Population

We studied 23 adult patients with mastocytosis (10 males and 13 females; age range: 29–76 years; median age 49 years) followed up at the University of Naples Federico II and at the University of Salerno.  Table 1 summarizes the patients' characteristics. None of the patients was on treatment for mastocytosis at the time of blood sampling. Twenty-three healthy individuals (10 males and 13 females; age range: 29–70 years; median age 43 years) were studied as the control group. Inclusion criteria were the absence of any known chronic or acute pathological condition at the time of enrollment, age > 18 years, ingestion of any anti-inflammatory and immunomodulating drugs at the time of the blood sampling, and expression of written informed consent. Exclusion criteria were the presence of any condition that, in the opinion of the investigator, could interfere with the completion of the study procedures and pregnancy.

Mediator-related symptoms were classified according to severity and frequency as follows: 6 patients had grade 0 (no symptoms), 7 had grade 1 (mild/infrequent: prophylaxis and/or as-needed therapy), 5 had grade 2 (moderate: kept under control with antimediator-type drugs daily), and 5 had grade 3 (severe and frequent: not sufficiently controlled with therapy). None of the patients had grade 4 characterized by a severe adverse event which requires immediate therapy and hospitalization [1].

The diagnosis and classification of mastocytosis were based according to the recommendation of the World Health Organization (WHO) on the histological examination of a skin biopsy for CM and of a bone marrow biopsy for SM [52]. Patients were divided according to cutaneous and/or systemic involvement and assessing the severity and frequency of symptoms. The first group (indolent) included maculopapular cutaneous mastocytosis (MPCM) (*n* = 2), mastocytosis in the skin (MIS) (*n* = 4), bone marrow mastocytosis (BMM) (*n* = 2), and indolent SM (ISM) (*n* = 7). The second group (advanced) included patients with smouldering SM (SSM) (*N* = 4), aggressive SM (ASM) (*N* = 3), and SM associated with hematologic disease (SM-AHD) (*N* = 1). The most common mutation of KIT receptors found in patients with indolent and aggressive SM is *KIT* D816V [53]. The assessment of *KIT* D816V mutation was performed in all patients with ASM (3 patients), SSM (5 patients), and SM-AHD (1 patient). Among patients with indolent mastocytosis, the assessment of *KIT* mutation was performed in those with high levels of tryptase (>100 ng/mL). Patient no. 14 and patient no. 21 show the presence of activating *KIT* mutation. We invited patients with provisional diagnosis of mastocytosis in the skin (4 patients) to undergo a bone marrow biopsy, but they refused. Lipid mediators, such as PLA_2_, are often lipoprotein-bound or associated with the circulation; therefore, lipid profile (cholesterol, low-density lipoprotein, high-density lipoprotein, and triglycerides) was assessed in all patients and controls. Three patients had a low level of cholesterol (84, 89, and 73 mg/dL, respectively); the remaining patients and controls had normal lipid profile.

### 2.2. Plasma Collection

The Ethical Committee Campania ASL Napoli 3 Sud (protocol number 68863) approved that plasma obtained during routine diagnostics could be used for research investigating the physiopathology of mastocytosis, and written informed consent was obtained from patients according to the principles expressed in the Declaration of Helsinki. The controls had been referred for routine medical check-up and volunteered for the study by giving informed consent. The samples were collected by means of a clean venipuncture and minimal stasis using sodium citrate 3.2%. In case of recent anaphylactic reactions, the measurement of all metabolites was performed at least two weeks after the acute event.

### 2.3. Tryptase

Plasma tryptase concentrations were measured by a fluoroenzyme immune assay (FEIA) using Uni-CAP100 (Phadia Diagnostics AB, Uppsala, Sweden). This technique allowed the measurement of both *α*-tryptase and *β*-tryptase. Normal values are 12.5 *μ*g/L.

### 2.4. Phospholipase Activity Assay

A modified liposomal-based fluorescent assay was used to measure PLA_2_ activity in plasma (Life Technologies EnzChek® phospholipase A_2_ assay). Results are expressed as units/L of PLA_2_ activity.

PLC activity was determined using the EnzChek® Direct Phospholipase C Assay kit (Life Technologies). Results are expressed as units/L of PLC activity.

PLD activity was assessed using a Sigma-Aldrich kit (catalogue number MAK137). This assay evaluates the hydrolysis of phosphatidylcholine to choline by PLD. Results are expressed as units/L of PLD activity.

### 2.5. Measurement of Endocannabinoids (AEA, 2-AG), *N*-Acylethanolamines (PEA, OEA), and DAGs

Plasma was sonicated and extracted with chloroform/methanol/Tris-HCl 50 mmol/L pH 7.5 (2 : 1 : 1, vol/vol) containing internal standards ([H_2_]8 AEA 5 pmol; [H_2_]5 2-AG, [H_2_]5 PEA, and [H_2_]4 OEA 50 pmol each) for EC quantification as well as 1,2-heptadecanoin (Larodan AB, Malmo, Sweden) for DAG measurement. The lipid-containing organic phase was dried down, weighed, and prepurified by open-bed chromatography on silica gel with 99 : 1, 90 : 10, and 50 : 50 (*v*/*v*) chloroform/methanol. The 90 : 10 fraction was used for EC and *N*-acylethanolamine quantification by LC-APCI-MS (LCMS-2020, Shimadzu) as previously reported [54]. DAG levels were measured by LC-MS-MS using an LC20AB coupled to a hybrid detector IT-TOF (Shimadzu Corporation, Kyoto, Japan) equipped with an ESI interface [55].

### 2.6. Statistical Analysis

Data were analyzed with the GraphPad Prism 5 software package. Data were tested for normality using the D'Agostino-Pearson normality test. If normality was not rejected at the 0.05 significance level, we used parametric tests. Otherwise, for not-normally distributed data, we used nonparametric tests. Statistical analysis was performed by an unpaired two-tailed *t*-test or two-tailed Mann-Whitney test as indicated in figure legends. Correlations between two variables were assessed by Spearman's correlation analysis and reported as coefficient of correlation (*r*). A *p* value ≤ 0.05 was considered statistically significant. Plasma levels of PLA_2_, PLC, DAGs, and ECs are shown as the median (horizontal black line), the 25^th^ and 75^th^ percentiles (boxes), and the 5^th^ and 95^th^ percentiles (whiskers) of 23 controls and 23 patients.

## 3. Results

### 3.1. PLA_2_ and PLC, but Not PLD, Plasma Activities Are Increased in Patients with Mastocytosis

**Figure 1:**
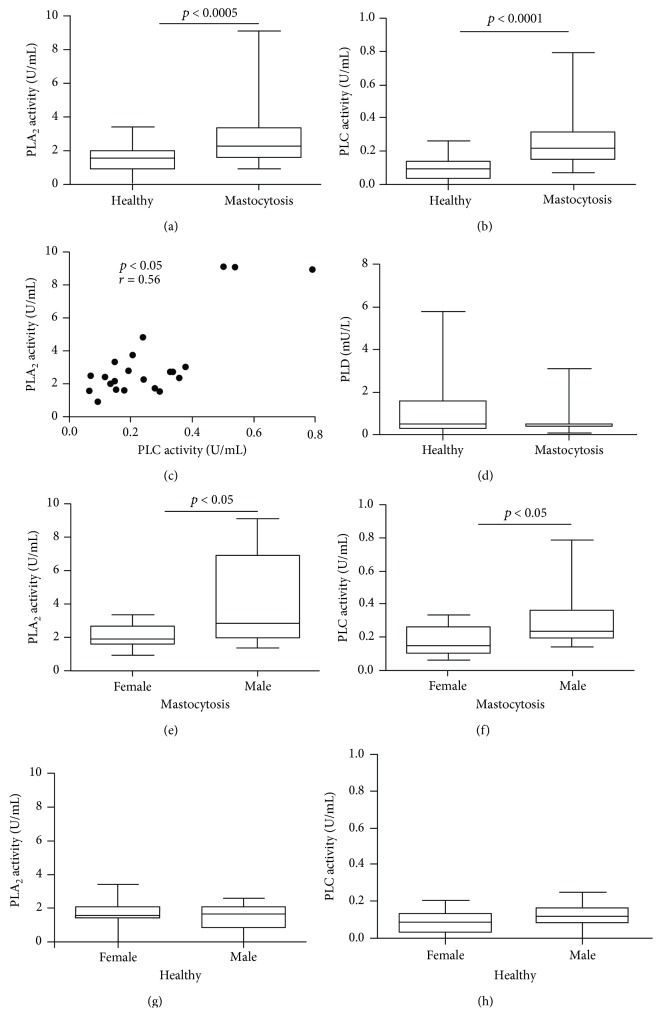
Activity of PLA_2_, PLC, and PLD in plasma of patients with mastocytosis and healthy controls. Data are shown as the median (horizontal black line), the 25^th^ and 75^th^ percentiles (boxes), and the 5^th^ and 95^th^ percentiles (whiskers) of 23 healthy controls and 23 mastocytosis patients for PLA_2_ (a), PLC (b), and PLD (d) assessment. Correlation between PLA_2_ and PLC (c) was assessed by Spearman's correlation analysis and reported as coefficient of correlation (*r*). PLA_2_ and PLC were measured in mastocytosis females and males (e, f) and healthy females and males (g, h).

We measured plasma PLA_2_, PLC, and PLD activities in patients with mastocytosis (*N* = 23) and age- and gender-matched healthy controls (*N* = 23) (Figure 1). Both PLA_2_ (Figure 1(a)) and PLC (Figure 1(b)) activities were increased in patients with mastocytosis compared to controls. There was a positive linear correlation between PLA_2_ and PLC activities (Figure 1(c)). By contrast, no difference in activity of PLD was found between patients and controls (Figure 1(d)).

There was no correlation between the age and the activity of PLA_2_ and PLC in both patients and controls (data not shown). PLA_2_ and PLC activities were higher in male mastocytosis patients (Figures 1(e) and 1(f)) whereas there was no gender difference in controls (Figures 1(g) and 1(h)).

PLA_2_, in particular group VII, are often lipoprotein-associated [22]. Only three of our patients had altered plasma cholesterol, but no correlation between lipid profile and PLA_2_ plasma activity was found in these patients (data not shown).

### 3.2. Increased DAG Concentrations in Patients with Mastocytosis

**Figure 2:**
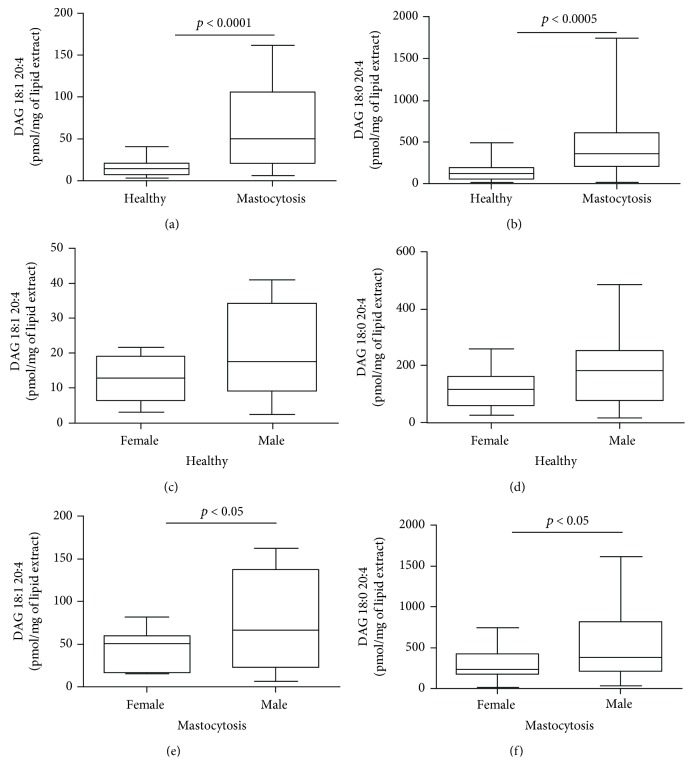
DAG 18:1 20:4 and 18:0 20:4 concentrations in plasma of patients with mastocytosis and healthy controls. DAG 18:1 20:4 (a) and DAG 18:0 20:4 (b) concentrations in healthy controls and mastocytosis patients. DAG 18:1 20:4 and DAG 18:0 20:4 concentrations in healthy females and males (c, d) and in mastocytosis females and males (e, f).

To evaluate whether the enhancement of PLC activity was accompanied by an increased production of DAGs, we measured DAG 18:1 20:4 and DAG 18:0 20:4 concentrations in the plasma of mastocytosis patients.  Figure 2 shows that both DAG 18:1 20:4 (a) and DAG 18:0 20:4 (b) concentrations in the plasma of mastocytosis patients were higher than in healthy controls. DAG 18:1 20:4 and DAG 18:0 20:4 concentrations were positively correlated with each other (Supplementary Figure 1). Like PLC, DAG concentrations did not correlate with the age of our study populations (data not shown) but were higher in male patients (Figures 2(e) and 2(f)). In mastocytosis patients, the concentrations of DAGs did not correlate with PLC activities (Supplementary Figures 2A-2B), suggesting that alternative sources of DAGs, or reduced DAG catabolism (see below), may occur in these patients or that phospholipid precursor availability, rather than PLC activity, is the limiting step for DAG biosynthesis.

### 3.3. Endocannabinoids in Patients with Mastocytosis

**Figure 3:**
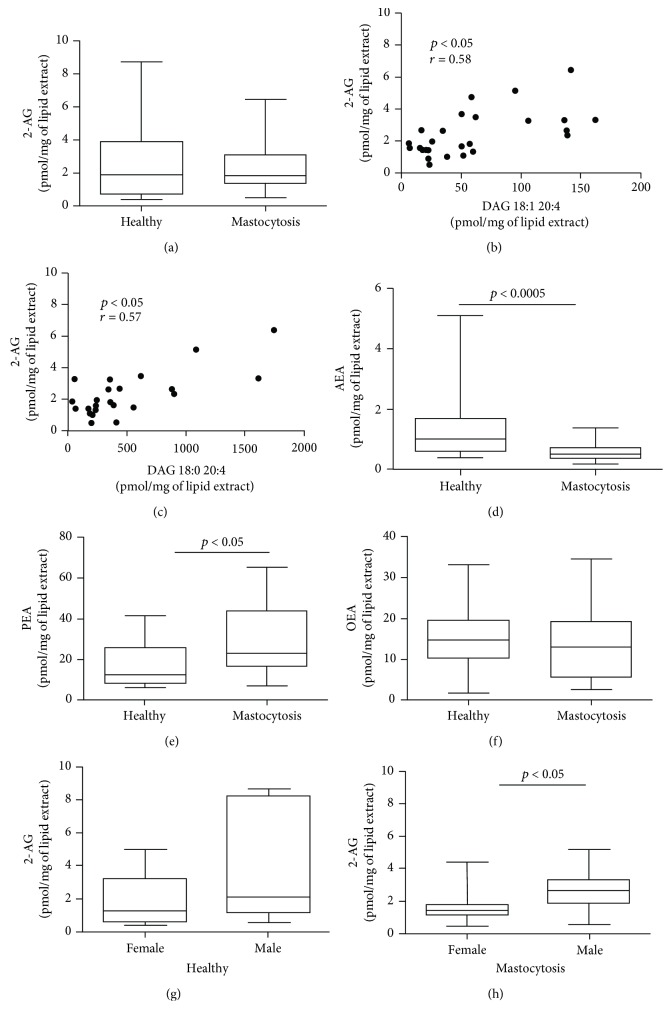
2-AG, AEA, PEA, and OEA concentrations in plasma of patients with mastocytosis and healthy controls. (a) 2-AG concentrations in healthy controls and mastocytosis patients. Correlation between 2-AG and DAG 18:1 20:4 (b) and DAG 18:0 20:4 (c) was assessed by Spearman's correlation analysis and reported as coefficient of correlation (*r*). AEA (d), PEA (e), and OEA (f) concentrations in healthy controls and mastocytosis patients. 2-AG concentration in healthy females and males (g) and in mastocytosis females and males (h).

Unlike the concentrations of its precursors (DAGs) (see above), 2-AG concentrations in patients with mastocytosis were similar to controls (Figure 3(a)), although they correlated positively with DAG concentrations (Figures 3(b) and 3(c)). Interestingly, AEA concentrations were lower in patients with mastocytosis compared to controls (Figure 3(d)). By contrast, PEA concentrations were increased in mastocytosis (Figure 3(e)). OEA concentrations did not differ between the two groups (Figure 3(f)).

No correlation was found between age and EC and *N*-acylethanolamine concentrations in either patients or healthy controls (data not shown). Males exhibited higher levels of 2-AG in both controls and patients compared to females (Figures 3(g) and 3(h)), whereas no gender differences were found in AEA and PEA concentrations (Supplementary Figures 3A-3F).

### 3.4. Relationships among PLA_2_, PLC, DAGs, and ECs and Disease Severity

**Figure 4:**
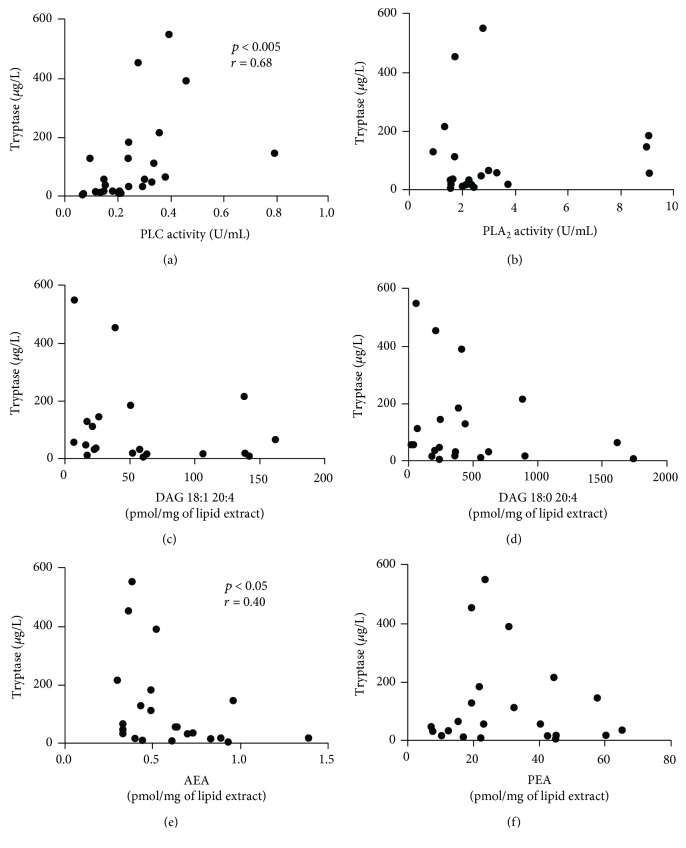
Relationships among PLC, PLA_2_, DAG, ECs, and tryptase concentrations. Correlations between two variables (PLC and tryptase (a), PLA_2_ and tryptase (b), DAG 18:1 20:4 and tryptase (c), DAG 18:0 20:4 and tryptase (d), AEA and tryptase (e), and PEA and tryptase (f)) were assessed by Spearman's correlation analysis and reported as coefficient of correlation (*r*). *p* value < 0.05 was considered statistically significant.

To understand whether altered concentrations of PLs and their metabolites reflected different degrees of disease severity, we used a multiple experimental analysis. First, we analyzed the correlation among lipid metabolites and tryptase because a significant proportion of patients with advanced forms of mastocytosis (ASM and SM-AHD) exhibit markedly elevated serum tryptase levels (often >200 mg/L) compared to those with ISM [44, 56]. PLC (Figure 4(a)), but not PLA_2_ (Figure 4(b)) and DAGs (Figures 4(c) and 4(d)), positively correlated with tryptase concentrations in mastocytosis patients. The concentrations of AEA, which are lower in patients (Figure 3(d)), negatively correlated with tryptase concentrations (Figure 4(e)). By contrast, the concentrations of PEA (Figure 4(f)) did not correlate with tryptase.

**Figure 5:**
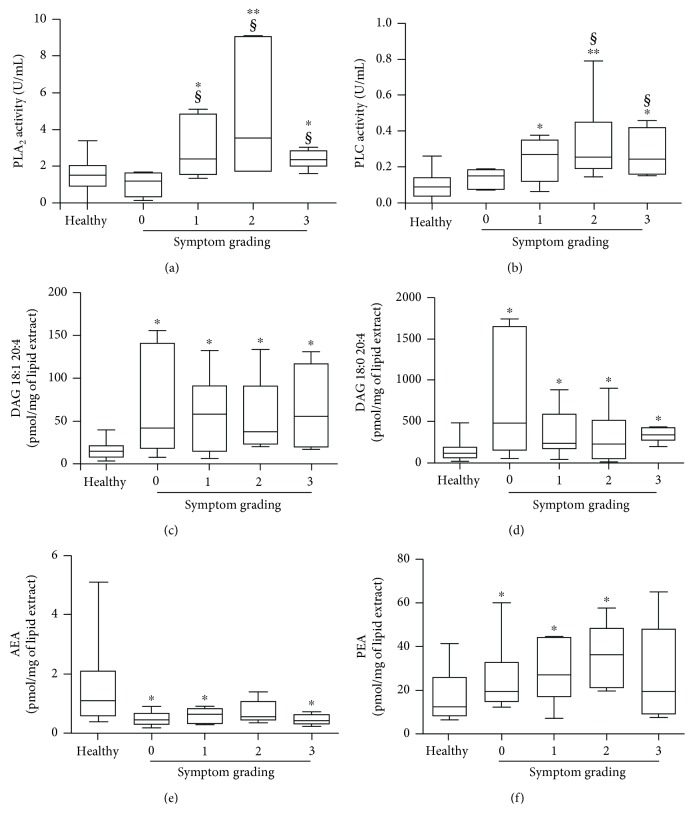
Relationships among PLA_2_, PLC, DAG, AEA, and PEA and symptom grading. PLA_2_ (a), DAG 18:1 20:4 (b), DAG 18:0 20:4 (c), PLC (d), AEA (e), and PEA (f) were determined in six patients with symptom grading 0, seven patients with grading 1, five patients with grading 2, and five patients with grading 3. ^∗^ *p* value < 0.05 and ^∗∗^ *p* value < 0.01 *vs.* healthy controls. ^§^ *p* value < 0.01 *vs.* patients with symptom grading 0.

Next, patients with mastocytosis were grouped according to the severity of mediator-related symptoms, and concentrations of PL, DAGs, AEA, and PEA levels were compared among groups. PLA_2_ and PLC activities were not increased in asymptomatic patients (grading 0) as compared to controls (Figures 5(a) and 5(b)). Patients with mediator-related symptoms (grading 1 to 3) had elevated PLA_2_ and PLC activities compared to both asymptomatic patients and healthy controls (Figures 5(a) and 5(b)). By contrast, DAG concentrations were increased in all groups of mastocytosis patients compared to controls (Figures 5(c) and 5(d)). AEA were generally lower (Figure 5(e)), and PEA were increased in all mastocytosis patients compared to controls, respectively (Figure 5(f)).

**Figure 6:**
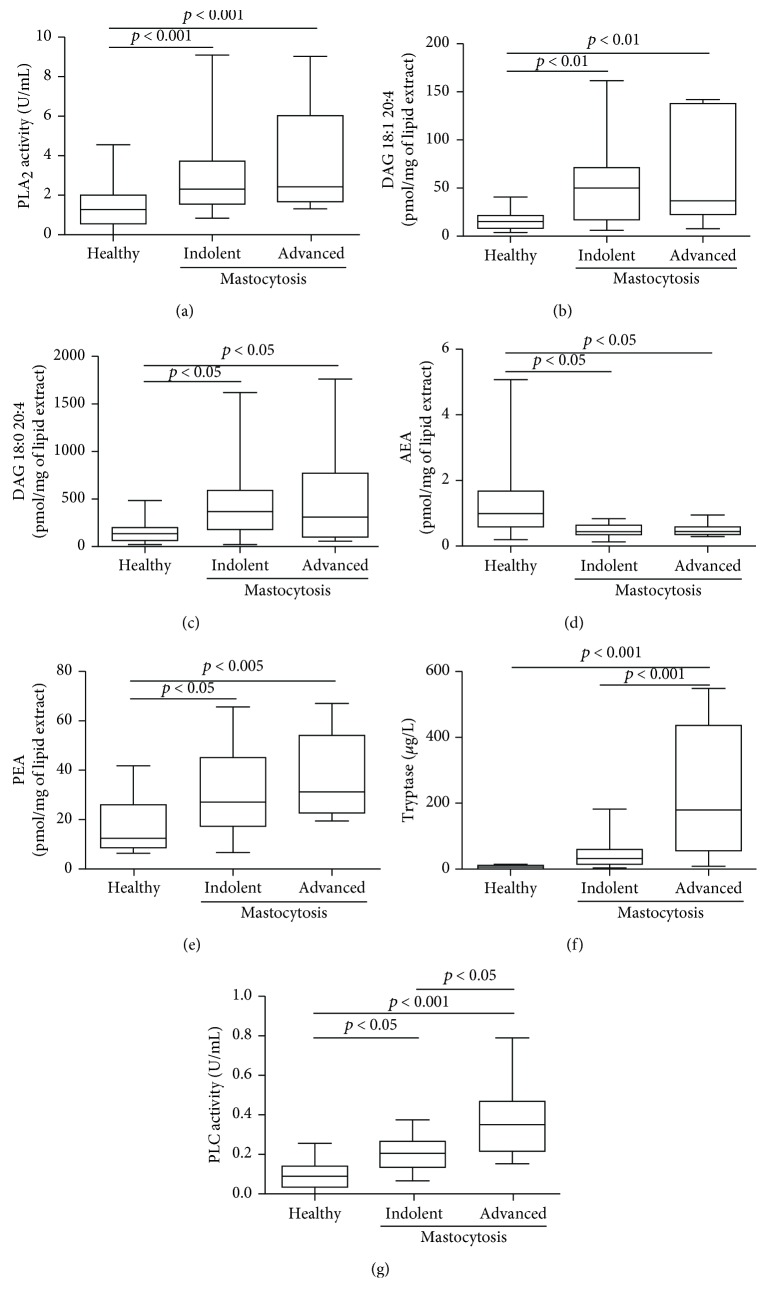
Relationships among PLA_2_, DAG, AEA, PEA, and PLC and mastocytosis clinical variants. PLA_2_ (a), DAG 18:1 20:4 (b), DAG 18:0 20:4 (c), AEA (d), PEA (e), tryptase (f), and PLC (g) were determined in healthy controls and in 15 patients with indolent variants and 8 patients with advanced variants.

Finally, we grouped patients according to their clinical variants in two groups (see Methods): indolent (MPCM/MIS/ISM/BMM) and advanced (SSM/SM-AHD/ASM) mastocytosis.  Figure 6 shows that PLA_2_ activities (Figure 6(a)), DAGs (Figures 6(b) and 6(c)), AEA (Figure 6(d)), and PEA (Figure 6(e)) concentrations did not differ between patients with indolent and advanced variants but were altered in both indolent and advanced variants when compared to controls. PLC activity, like tryptase, was higher in patients with advanced mastocytosis compared to indolent variants (Figure 6(f)), but unlike tryptase, PLC activities were also increased in indolent mastocytosis compared to controls (Figure 6(g)).

## 4. Discussion

In this study, we describe for the first time that plasma PL activities and concentrations of their metabolites (e.g., DAGs and 2-AG) are significantly altered in patients with mastocytosis. Patients with mastocytosis have (1) increased plasma activities of PLA_2_ and PLC, (2) elevated DAGs and PEA concentrations, and (3) decreased levels of AEA. It is well known that PLs control MC degranulation [9, 35] and eicosanoid production, two conditions associated with mastocytosis [31–34]. Antagonists and/or inhibitors of synthesis of eicosanoids are currently used to treat mediator-related symptoms in patients with mastocytosis [57, 58]. These observations are in line with the results of our study showing that some of these molecules, in particular PLs, are significantly increased in patients with more severe symptoms and disease phenotype.

Mastocytosis is caused by an activating mutation of *KIT* that leads to uncontrolled proliferation and accumulation of MCs with heterogeneous clinical manifestations ranging from cutaneous and advanced forms with poor prognosis [3, 4]. Our results suggest that PLA_2_ and PLC could be involved in the development of mediator-related symptoms in patients with mastocytosis. In fact, PLA_2_ and PLC activities are increased in symptomatic but not in asymptomatic patients when compared to healthy controls. These data are consistent with the known effects of PLA_2_ and PLC on MCs. Indeed, some evidence demonstrates the role of PLA_2_ in MC activation through cPLA_2_ involvement. Kikawada and coworkers reported that in MCs lacking PLA_2_ group V, the time course of phosphorylation of ERK 1/2 and cPLA_2_ was markedly decreased, leading to attenuation of eicosanoid formation in response to stimulation through TLR2 but not through c-kit or Fc*ε*RI [59]. Phospholipase C- (PLC-) *β*3 is crucial for Fc*ε*RI-mediated MC activation [35]. MCs are a source and target of sPLA_2_, in particular, of group IIA (PLA2G2A) and groups V (PLA2G5) and III (PLA2G3) [9, 18]. Overexpression of PLA2G2A in rat MCs augments degranulation [9, 17] and triggers histamine [30] and PGD_2_ release [60], whereas overexpression of PLA2G3 leads to spontaneous skin inflammation [9, 61, 62].

Secretory phospholipases are increased in biological fluids of patients with several disease such as inflammatory, cardiovascular, and autoimmune diseases and cancer [23, 63–67]. In this study, we have not assessed the specific PLA_2_ group(s) secreted in mastocytosis; however, it is reasonable to hypothesize that PLA2G2A, which is the major secreted form of PLA_2_ in human serum and plasma [23, 63–67], is responsible for most of the detected PLA_2_ activity in mastocytosis. A time-resolved fluoroimmunoassay (TR-FIA) on plasma and confocal microscopy analysis of tissue biopsies could identify the existence of types of PLA_2_ involved in mastocytosis.

Tryptase is the most widely used circulating marker of mastocytosis [68, 69] and is also an easy accessible predictor for disease progression in patients with indolent mastocytosis [56, 68]. Our results show that most patients with advanced forms of mastocytosis have markedly increased plasma PLC activities compared to those with indolent forms. In addition, PLC activities were positively correlated with tryptase concentrations. It will be interesting to evaluate whether the plasma levels of this enzyme at time of diagnosis could predict the clinical severity of mastocytosis.

Several PLC products such as DAG 18:1 20:4 and DAG 18:0 20:4 are increased in patients with mastocytosis, but their concentrations are similar in indolent and advanced variants. Interestingly, DAG concentrations are positively correlated with those of their metabolite 2-AG, even though 2-AG concentrations are not altered. Other sources of DAGs and/or alternative biosynthetic precursors for 2-AG, rather than shortage of DAGL activity, might explain this finding. It is conceivable that the increased DAG concentrations in mastocytosis reflect altered PKC activation, essential for release of preformed mediators in MC granules [36], rather than the production of 2-AG, which by activating CB_2_ cannabinoid receptors would instead counteract this effect [40].

In addition to previously discovered molecules aimed at controlling cellular (MC) activation, *N*-acylethanolamines (for example, AEA and its congener PEA) are involved in endogenous, cannabinoid receptor-dependent and independent, protective mechanisms that are activated as a result of different types of tissue damage or stimulation of inflammatory responses and nociceptive fibers [70]. We mentioned above the large body of evidence indicating that PEA has anti-inflammatory actions and inhibits MC degranulation [40]. Thus, the increase of PEA plasma levels in mastocytosis could represent an attempt to control the activation of MCs. By contrast, the decrease of AEA concentrations and its negative correlation with tryptase levels may contribute to the underlying inflammation associated with this disorder.

Human tryptase is considered highly specific of MCs, which may contain high amounts, up to 35 pg per cell [71–73]. Although basophils may produce small quantities of tryptase, the vast majority of tryptase in the blood is derived by MCs [74, 75]. Detection of tryptase provides information about MC distribution, numbers, proliferation, and activation status [76] and is, therefore, a major marker of mast cell disorders, including mastocytosis [44, 56, 77, 78].

Unlike tryptase, PLs and the metabolites measured in this study are produced not only from MCs but also from other leukocytes such as neutrophils, eosinophils, and macrophages [9, 18, 41, 79–81]. The biologic activity of PLs is not confined to MCs but includes other immune and nonimmune cells [41, 82–84]. Our data show that both PLA_2_ and PLC are increased in plasma of patients with mastocytosis and that there is a correlation between PLC activity and serum tryptase but not between PLA_2_ and tryptase. These results indicate that these enzymes are secreted by cells that are activated in mastocytosis, but they do not allow to discriminate whether they are released from MCs or by other cells that could be indirectly activated in these patients. On the other hand, the cellular sources of PLs, DAGs, and PEA in the plasma of patients with mastocytosis are unknown, and further studies are needed to understand the origin of these enzymes in these patients.

It has been shown that the *KIT* activation generates PLC signal, DAG formation, and PKC activation [85–87]. This study shows an increase of PLs in patients with more symptoms and with advanced form of mastocytosis. A question that remains to be answered is whether activating mutations of *KIT* lead to an abnormal PL activation that could contribute to the development of symptoms and to increase severity of mastocytosis. Future studies will compare the levels of PLs and their metabolites in patients with and without *KIT* mutation.

In conclusion, we demonstrate that plasma levels of PLs, DAGs, and some *N*-acylethanolamines are altered in patients with mastocytosis and that PLC activity is further increased in patients with symptomatic and aggressive forms of disease. These results suggest a relevant but different and, in some cases, opposing role of these mediators in mastocytosis. Further studies are needed to evaluate the diagnostic and prognostic value of PLs, DAGs, and *N*-acylethanolamines in different forms of mastocytosis and to understand whether pharmacological blockade of these molecules (e.g., PKC) may improve the symptoms and severity of mastocytosis.

## Figures and Tables

**Table 1:** Characteristics of 23 adult patients with mastocytosis.

Patient no.	Sex	Age	Disease category	Tryptase (*μ*g/L)	Symptom grading
1	F	38	MIS	17.2	0
2	F	49	BMM	17.8	0
3	M	49	BMM	32.6	0
4	M	62	ISM	64.5	0
5	M	66	SSM	551	0
6	F	33	SM-AHD	7.9	0
7	F	29	MPCM	11.5	1
8	F	49	MPCM	5	1
9	F	26	MIS	15.8	1
10	F	45	MIS	47.6	1
11	M	50	MIS	127	1
12	M	54	SSM	216	1
13	M	57	ISM	58.4	1
14	F	42	ISM	184	2
15	F	49	ISM	56.5	2
16	M	71	ISM	17.6	2
17	M	50	SSM	454	2
18	F	57	SSM	112	2
19	M	35	ASM	145	2
20	F	35	ISM	32.4	3
21	F	55	ISM	129	3
22	F	45	ASM	36.2	3
23	M	76	ASM	390	3

ASM: aggressive systemic mastocytosis; BMM: bone marrow mastocytosis; ISM: indolent systemic mastocytosis; MIS: mastocytosis in the skin; MPCM: maculopapular cutaneous mastocytosis; SM-AHD: systemic mastocytosis associated with hematologic disease; SSM: smouldering systemic mastocytosis.

## Data Availability

All data are fully available without restriction. All relevant data are within the paper and its supporting information files.

## Abbreviations

AEA: Anandamide
AA: Arachidonic acid
ASM: Aggressive systemic mastocytosis
2-AG: 2-Arachidonoylglycerol
BMM: Bone marrow mastocytosis
CM: Cutaneous mastocytosis
DAGs: Diacylglycerols
DAGLs: DAG lipases
ECs: Endocannabinoids
FEIA: Fluoroenzyme immune assay
ISM: Indolent systemic mastocytosis
MCs: Mast cells
MPCM: Maculopapular cutaneous mastocytosis
MIS: Mastocytosis in the skin
PLs: Phospholipase enzymes
PEA: Palmitoylethanolamide
PGD_2_: Prostaglandin D_2_
PKC: Protein kinase C
OEA: Oleoylethanolamide
sPLA_2_s: Secreted PLA_2_
SSM: Smouldering systemic mastocytosis
SM: Systemic mastocytosis
SM-AHD: Systemic mastocytosis associated with ematologic disease
WHO: World Health Organization.

## Supplementary Materials

Supplementary Materials Supplementary Table 1: the variants of systemic mastocytosis (SM). Supplementary Figure 1: the correlation between DAG 18:1 20:4 and DAG 18:0 20:4 in plasma of patients with mastocytosis. Supplementary Figure 2: the correlation between PLC and DAGs in plasma of patients with mastocytosis. Supplementary Figure 3: the relationship between AEA, PEA, and OEA concentrations and gender of patients with mastocytosis and healthy controls.
