# Supplementary material for: Altered Metabolism of Phospholipases, Diacylglycerols, Endocannabinoids, and N-Acylethanolamines in Patients with Mastocytosis
Source: J Immunol Res. 2019 Jul 1;2019:5836476. doi: 10.1155/2019/5836476 (PMC6636572; doi:10.1155/2019/5836476)
Supplement: Supplementary Materials — Supplementary Table 1: the variants of systemic mastocytosis (SM). Supplementary Figure 1: the correlation between DAG 18:1 20:4 and DAG 18:0 20:4 in plasma of patients with mastocytosis. Supplementary Figure 2: the correlation between PLC and DAGs in plasma of patients with mastocytosis. Supplementary Figure 3: the relationship between AEA, PEA, and OEA concentrations and gender of patients with mastocytosis and healthy controls. [file 5836476.f1.docx]

Supplementary Table 1. WHO classification of mastocytosis 2016 (modified from Valent P. et al., Blood 2017) (4)

| Variant | Abbreviation |
| --- | --- |
| Cutaneous Mastocytosis   - Maculopapular CM - Diffuse CM   Systemic Mastocytosis   - Indolent SM - Smouldering SM - SM associated to Hematologic Disease - Aggressive SM | CM  MPCM  DCM  ISM  SSM  SM-AHD  ASM |
| - Mast Cell Leukemia | MCL |
| MC Sarcoma |  |

**Supplementary Figure 1. Correlation between DAG 18:1 20:4 and DAG 18:0 20:4 in plasma of patients with mastocytosis.**

Correlations between two variables: DAG 18:1 20:4 and DAG 18:0 20:4 were assessed by Spearman’s correlation analysis and reported as coefficient of correlation (r).


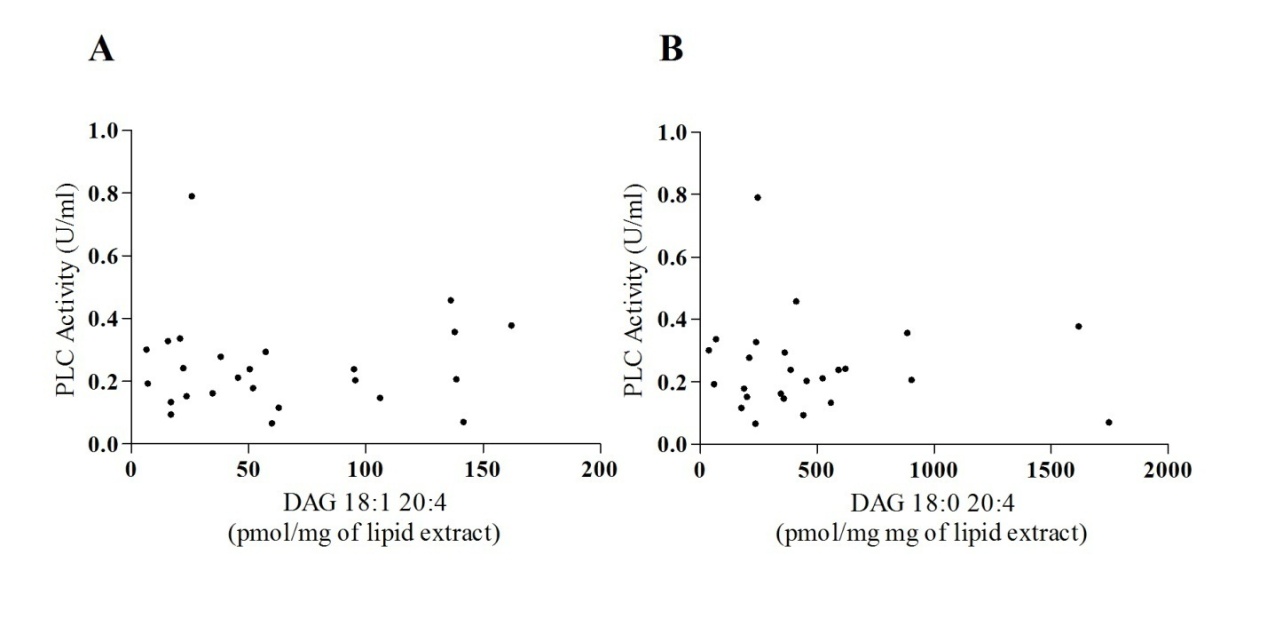


**Supplementary Figure 2. Correlation between PLC and DAGs in plasma of patients with mastocytosis.**

Correlations between two variables: PLC and DAG 18:1 20:4 (A) and PLC and DAG 18:0 20:4(B) were assessed by Spearman’s correlation analysis and reported as coefficient of correlation (r).


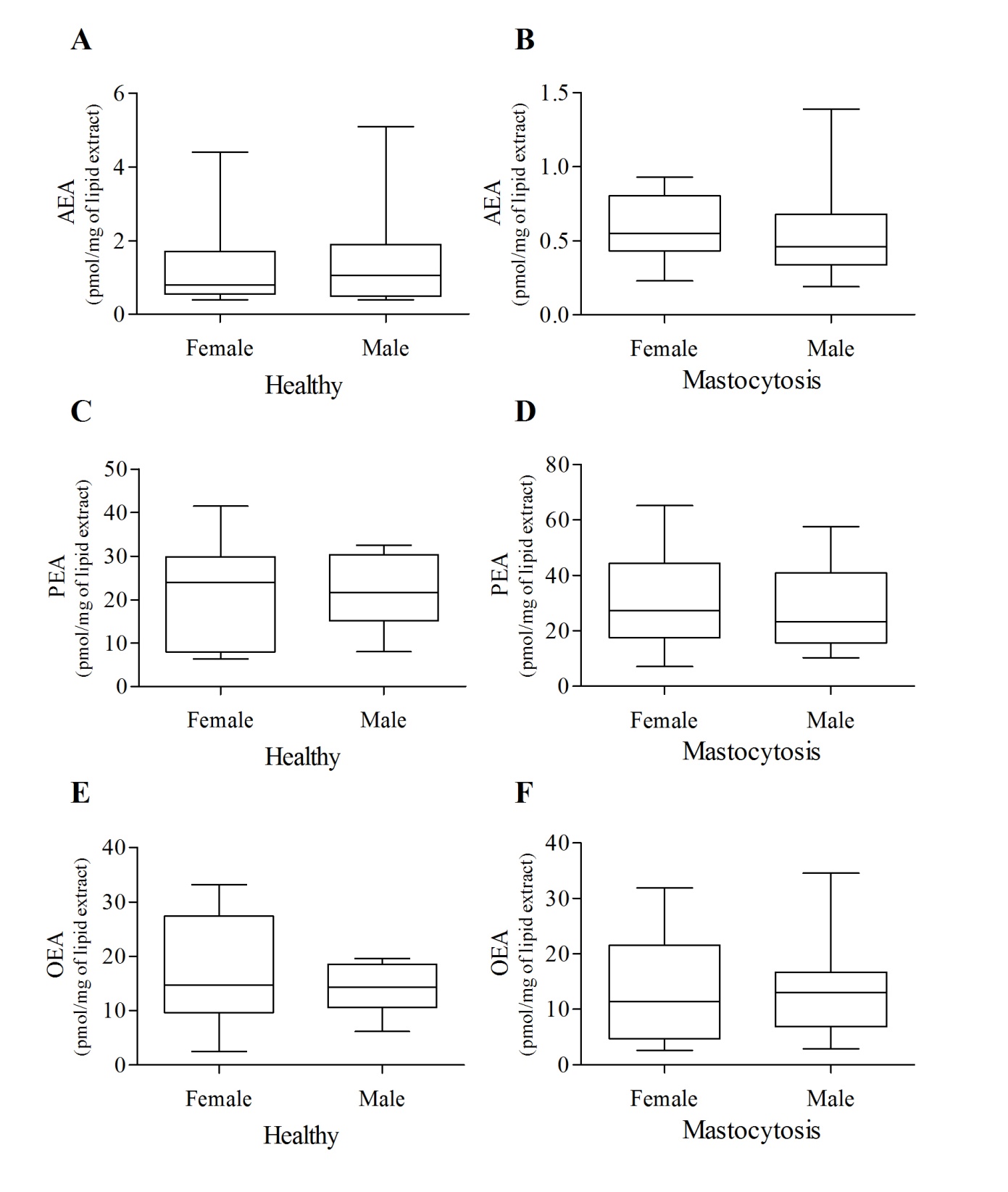


**Supplementary Figure 3. Relationship between AEA, PEA and OEA concentrations and gender of patients with mastocytosis and healthy controls.**

AEA (A-B), PEA (C-D) and OEA (E-F) were measured by LC-MS analysis in healthy females (n.13) and males (n. 10) (A, C, E) and in mastocytosis females (n. 13) and males (n. 10) (B, D, F).
